# ViDscribe: Multimodal AI for Customizing Audio Description and Question Answering in Online Videos

**DOI:** 10.1145/3772363.3798744

**Published:** 2026-04-13

**Authors:** Maryam S Cheema, Pooyan Fazli, Sina Elahimanesh, Hasti Seifi

**Affiliations:** Arizona State University Tempe, Arizona, USA; Arizona State University Tempe, Arizona, USA; Saarland University Saarbrücken, Saarland, Germany; Arizona State University Tempe, Arizona, USA

**Keywords:** Video Accessibility, Blind and Low Vision Users, Audio Description, Multimodal Large Language Models, Customization, Visual Question Answering, Longitudinal Study

## Abstract

Advances in multimodal large language models enable automatic video narration and question answering (VQA), offering scalable alternatives to labor-intensive, human-authored audio descriptions (ADs) for blind and low vision (BLV) viewers. However, prior AI-driven AD systems rarely adapt to the diverse needs and preferences of BLV individuals across videos and are typically evaluated in controlled, single-session settings. We present *ViDscribe*, a web-based platform that integrates AI-generated ADs with six types of user customizations and a conversational VQA interface for YouTube videos. Through a longitudinal, in-the-wild study with eight BLV participants, we examine how users engage with customization and VQA features over time. Our results show sustained engagement with both features and that customized ADs improve effectiveness, enjoyment, and immersion compared to default ADs, highlighting the value of personalized, interactive video access for BLV users.

## 1 Introduction

Recent advances in multimodal large language models (MLLMs) enable automated audio descriptions (AD) and interactive question answering for video content [22, 32, 37], creating new opportunities to improve access for blind and low vision (BLV) users. While human-produced AD can convey rich narrative detail, capturing visual nuance, emotional tone, and complex scene dynamics [4], it is costly, time-consuming, and requires specialized expertise. As a result, the vast majority of online video content remains undescribed [25]. In response, prior work suggests that AI-generated AD can provide effective and scalable access to visual media for BLV users [8, 22, 34].

Despite this promise, existing AI-generated AD systems largely adopt a one-size-fits-all approach and are typically evaluated in short, lab-based studies with preset videos. Recent research highlights the importance of customization [8, 16] and interactive question answering [18] for supporting BLV users’ diverse information needs and viewing goals. However, prior customization efforts focus primarily on human-produced or edited descriptions [24]. Little is known about how well MLLMs can support on-demand AD customization and video question answering (VQA) for online videos. Moreover, prior evaluations of AI-generated AD rarely consider longitudinal use. Consequently, we lack understanding of how BLV users’ interaction patterns, preferences, and perceptions of AI-generated descriptions evolve over time in everyday video-watching contexts.

**Figure 1:**
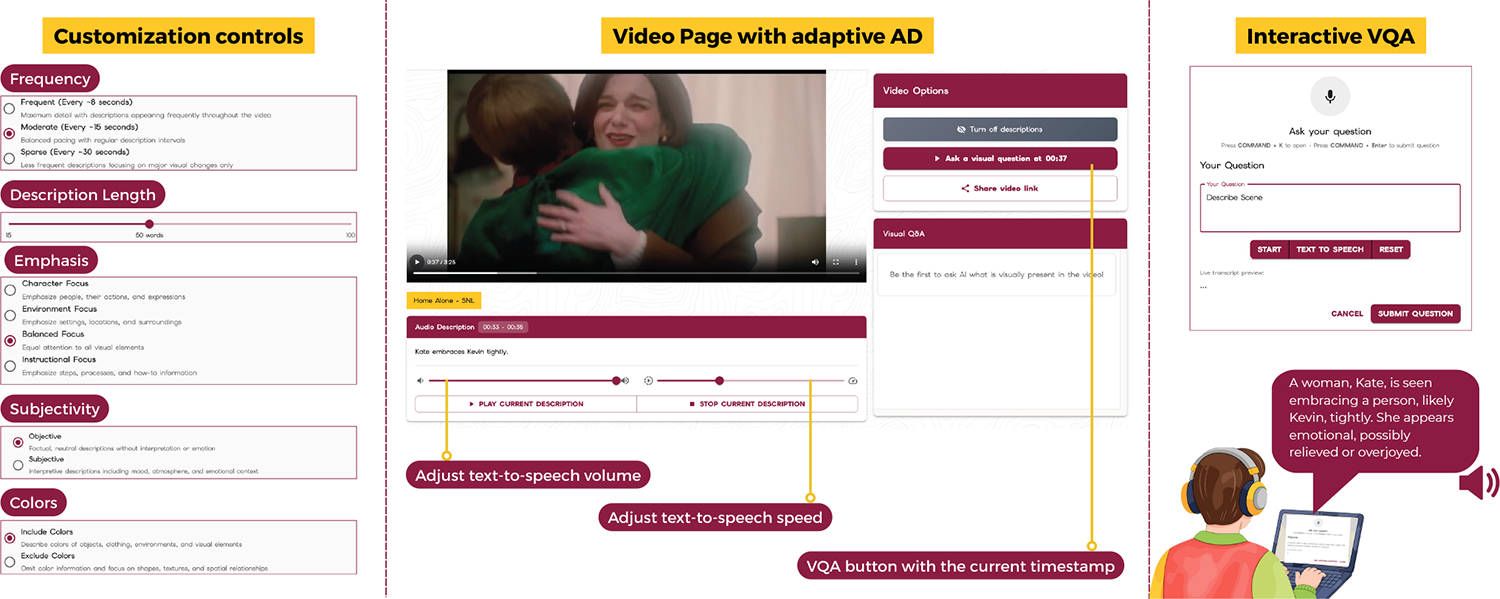
*ViDscribe* interface, showing customization controls, video page with adaptive ADs, and interactive VQA during playback.

To address these gaps, we develop *ViDscribe*, an online platform that provides AI-generated AD, multiple forms of user-driven AD customizations, and an integrated question-and-answer feature (Figure 1). *ViDscribe* supports any YouTube video, enabling BLV users to engage with content they independently select rather than researcher-curated media. Using this platform, we investigate three research questions: **(1)** How well can MLLMs support AD customization for BLV users? **(2)** What types of customizations and questions do BLV users most frequently request while watching online videos? and **(3)** How do patterns of AD customization and VQA change over time during repeated, everyday use?

To answer these questions, we conduct a longitudinal user study with eight BLV participants. Each participant completes at least five video-viewing sessions with *ViDscribe* over a week. The study combines interaction logs with in-situ daily micro-surveys and post-study feedback to capture both immediate reactions and evolving usage behaviors. Our findings show that BLV users frequently customized ADs, reporting higher effectiveness, enjoyment, and immersion than with defaults. We report the most frequent types of VQA queries and AD customizations, as well as a shift over time toward shorter and less frequent descriptions, reflecting evolving user preferences.

This work makes two contributions:

- Design and implementation of *ViDscribe*^1^, an online platform that leverages MLLMs to provide automated AD, user-driven customization, and interactive VQA for arbitrary online videos; and
- Empirical data from a longitudinal study that characterizes BLV individuals’ usage patterns, preferences, and perceptions of AI-generated AD customizations and VQA in everyday video-watching contexts.

## 2 Related Work

Our work lies at the intersection of interactive audio description tools for video accessibility and research on video personalization and question answering for BLV users.

### Audio Description Tools.

A wide range of tools support AD creation, from manual platforms such as YouDescribe [31], Re-scribe [28], and Omnidescribe [7], to AI-based approaches including SPICA [27] and NarrationBot [5, 15], which enable automatic generation of video descriptions [6, 22, 34, 35]. Hybrid methods combine human expertise with automation to produce timely, contextually relevant descriptions [9, 26]. For instance, DescribePro leverages MLLMs to assist novice and professional describers in creating and collaborating on adaptive and style-aware ADs [9]. While human-in-the loop tools capture rich emotional and contextual nuance [38, 39], they cannot scale to the increasing volume of video content [23]. Recent advances in large language and vision models accelerate AD generation [12] and produce higher-quality descriptions than novice human-generated ADs [21, 22]. Yet, most systems still produce a single, fixed description per video, limiting responsiveness to various content and individual preferences.

### Personalized ADs and Video QA.

BLV users have diverse needs shaped by viewing context [8, 16, 33, 36], and degree of blindness [10], highlighting the importance of personalization and interactivity in accessible video experiences [30]. Prior work shows that professionally customized ADs can significantly improve BLV users’ comprehension, satisfaction, and engagement [11, 24], highlighting the potential for scalable, automated customization [24]. Beyond passive descriptions, interactive question answering emerges as a complementary approach that enables users to request missing details and clarify ambiguities on demand [2, 5, 17, 19], thus enhancing agency and learning [1]. Yet prior work rarely examines these features in real-world or longitudinal contexts. Building on this literature, our work investigates BLV users’ week-long experience with AI-generated customized ADs combined with VQA, providing insights into how personalization and interactivity shape everyday video accessibility.

## 3 ViDscribe

*ViDscribe* is a web-based, AI-powered platform designed to support BLV users through adaptive AD and interactive VQA (Figure 1). Users can watch any YouTube video by pasting its URL, and optionally selecting AD customization settings before playback. Based on these inputs, *ViDscribe* generates personalized ADs that are synchronized with the video, while allowing users to ask questions at any point during playback. Users can also adjust the speed and volume of the AD speech while watching. *ViDscribe* is implemented using a React frontend and AWS Lambda for the backend. The system leverages the *Gemini 3 Pro* multimodal large language model for both AD generation and visual question answering. The interface is fully compatible with screen readers and supports keyboard-based controls for playback, customization, and AD speech-rate adjustment.

### (1) Customization Controls.

To accommodate diverse preferences and viewing contexts, *ViDscribe* provides six customization options informed by prior work [8, 9, 24] and our experience with BLV users. These settings can be configured per video and adjusted as needed: **(A) Frequency:** Controls how often audio descriptions are inserted, including frequent (every 8 seconds), moderate (15s), or sparse (30s); **(B) Length:** Adjusts the number of words per description using a slider (15–100 words); **(C) Emphasis:** Prioritizes the type of visual information described, such as general content, characters, instructional, or environment focus; **(D) Subjectivity:** Switches between objective, factual descriptions and more subjective, interpretive narration; **(E) Color Preference:** Toggles whether color attributes are explicitly described **(F) Free-form Guidelines:** Allows users to optionally enter custom instructions via a textbox to guide AD generation further. All customization settings are translated into prompt parameters to condition AD generation. **Default AD** is produced by our system when participants choose not to select any customization options. It applies these preset parameters: a description length of 50 words, general emphasis with objective descriptions, and color attributes enabled. This approach enables BLV users to access any YouTube video, including those without a human-written AD track.

### (2) Adaptive AD Generation.

*ViDscribe* separates AD timing from content generation to ensure temporal alignment and intelligibility. First, the AD timing module identifies suitable timestamps for inserting descriptions by following established AD guidelines and adapting the DescribePro approach [9]. The module extracts the video’s audio track and visual frames, then analyzes three signals: (1) silence, (2) no-speech segments, and (3) scene changes. Silence indicates the absence of sound, while no-speech captures moments without human speech, helping avoid interruptions to dialogue. Scene change detection ensures that ADs align with meaningful visual transitions. The module selects timestamps where these signals overlap, prioritizing natural pauses that coincide with visual changes, and recursively splits intervals longer than the user-selected frequency to allow multiple AD insertions when appropriate. Next, the description generation module uses *Gemini 3 Pro* to generate ADs for each timestamped segment. The system provides the model with the video, the timestamps, the user’s customization settings, and a set of 42 AD guidelines from prior work [22]. *Gemini 3 Pro* then produces descriptions tailored to the requested frequency, length, emphasis, subjectivity, and color preferences. Full prompts and guidelines are included in Supplementary Materials.

### (3) Interactive VQA Feature.

Beyond passive listening, *ViDscribe* supports interactive visual question answering during video playback. By pressing a dedicated keyboard shortcut or on-screen button, users can ask questions (e.g., “Who just entered the room?”) either by typing or using speech-to-text. To answer a question, the system sends the user’s query along with the current video timestamp, video AD, and representative video frames to *Gemini 3 Pro*, which generates a concise, context-aware response. Answers are presented as text and rendered via text-to-speech, enabling on-demand clarification and deeper engagement with visual content during everyday video watching.

## 4 User Study

We conducted a five-day study with eight BLV users to examine patterns of AD customization and VQA with *ViDscribe* outside the lab. The study was approved by the university’s ethics board. Participants received $75 for their time.

### Participants.

We recruited eight participants (4 female, 3 male, 1 non-binary; *M*_*age*_ = 39.8). All participants identified as blind (totally blind = 4, light perception = 3, legally blind = 1) and used screen readers. Four participants reported frequently watching videos with ADs, three occasionally, and one rarely. Seven participants had prior interaction with AI descriptions, either through user studies (*n* = 4), or using visual description apps (e.g., PiccyBot, SeeingAI).

### Study Procedure.

The study consisted of (1) an introductory session, (2) a five-day usage period over a week, and (3) an end-of-week survey. In the introductory session (20–30 minutes), participants provided consent, completed a demographics questionnaire, and were introduced to *ViDscribe*. Participants then used the system to watch at least 10 minutes of video per day for at least five days at times and locations of their choice. After each day, they completed a short survey about their experience, followed by a final survey at the end of the week.

### Data Collection.

In end-of-day surveys, participants rated the effectiveness, enjoyment, and immersion of watching videos with *ViDscribe*. When used, they also rated customization alignment, helpfulness, and accuracy of VQA responses. The final survey included System Usability Scale (SUS) scores. We analyzed VQA questions using a closed-code codebook developed by one author and independently applied by two authors. For each question, both authors reviewed the video at the timestamp when the question was asked to determine whether the system’s response was accurate and answerable based on the visual information. Disagreements in the coding for VQA types and accuracy were resolved through discussion. We analyzed open-ended survey responses using open coding. The system logged uploaded videos and user customizations.

## 5 Results

Over the longitudinal study, participants uploaded 81 videos (average duration = 3:37), 51 of which used customizations (i.e., 63%). They completed 45 daily surveys (i.e., diary entries), including 31 customization-alignment ratings and 26 VQA ratings. Below, we report findings on their experience, customization and VQA usage, and changes over time.

### User Experience.

**Figure 2:**
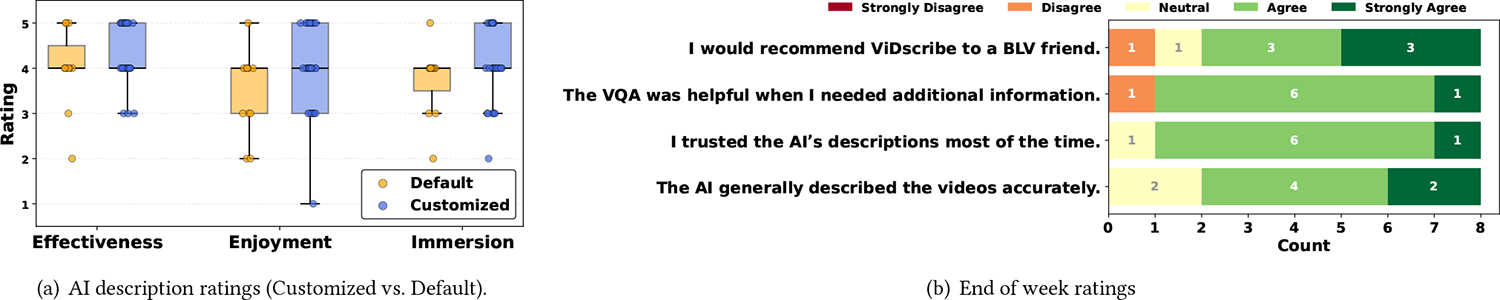
User ratings for (a) customized vs. default ADs in daily surveys, and (b) *ViDscribe* in the end-of-week survey.

AI customizations received higher effectiveness, enjoyment, and immersion ratings than under default settings (Fig. 2(a)). Enjoyment showed the largest increase (*M*_*custom*_= 3.97; *M*_*default*_ = 3.45), echoed in qualitative feedback: *[P5] “the last video was actually quite enjoyable…I could see myself actually using this.”* Ratings for immersion(*M*_*custom*_ = 4.06; *M*_*default*_ = 3.72) and effectiveness (*M*_*custom*_ = 4.32; *M*_*default*_ = 4.00) showed similar trends. Due to the small sample size and unequal numbers of observations, we did not conduct statistical significance tests.

Ratings for the VQA feature were positive but comparatively lower. Participants asked a total of 66 questions across the study. In the final survey, participants rated VQA particularly useful when seeking additional information (Fig 2(b)). In daily surveys, participants rated how much VQA helped them understand videos as 3.46 out of 5, while perceived accuracy was rated slightly lower at 3.27. Based on our manual analysis of VQAs, the lower ratings likely stem from cases in which participants (*n* = 2) needed to reframe or rewind videos to ask their questions, as the current VQA implementation uses only the current frame and a few adjacent frames to provide an answer.

Overall, *ViDscribe* received an average SUS score of 70.6 (*SD* = 19.4), above the average benchmark for web-based interfaces of 68 [3]. Participants suggested improving keyboard navigation, particularly for accessing VQA (*n* = 3). Additional feedback highlighted a desire to save customization and text-to-speech settings for future use. The end-of-week ratings suggested trust in the AI-generated descriptions, and most participants (*n* = 6) indicated they would recommend the system to others (Fig. 2(b)).

### Types of Customizations and Questions.

Table 1 summarizes the AD customization options selected across videos. Participants more frequently opted for general emphasis and objective descriptions. However, character focus was popular for *Sports* (3/5) and *Film & Animation* (4/14) videos. The use of free-form guidelines was also higher in *Sports* (2/5) and *Film & Animation* (5/12), where many of these guidelines concerned character attributes. For example, requests were made to include character names (*“Include character names”*, n=5) or to describe appearance (*“Describe the apparent age and physical health of the people in the video,”* n=2). This aligns with the preference for character emphasis for *Film & Animation* genre. Compared to other genres, *Film & Animation* and *Entertainment* videos showed greater use of subjective descriptions (5/14 and 3/5, respectively), suggesting varied preferences for visually rich content. Instructional emphasis was particularly popular for *How-To videos* (6/11): *“The instruction emphasis is very awesome for educational, crafts, and exercise videos (P7).”* Other than one instance, no videos used the 30-second frequency setting, and most videos used descriptions under 50 words, indicating a general preference for shorter, more frequent descriptions.

**Figure 3:**
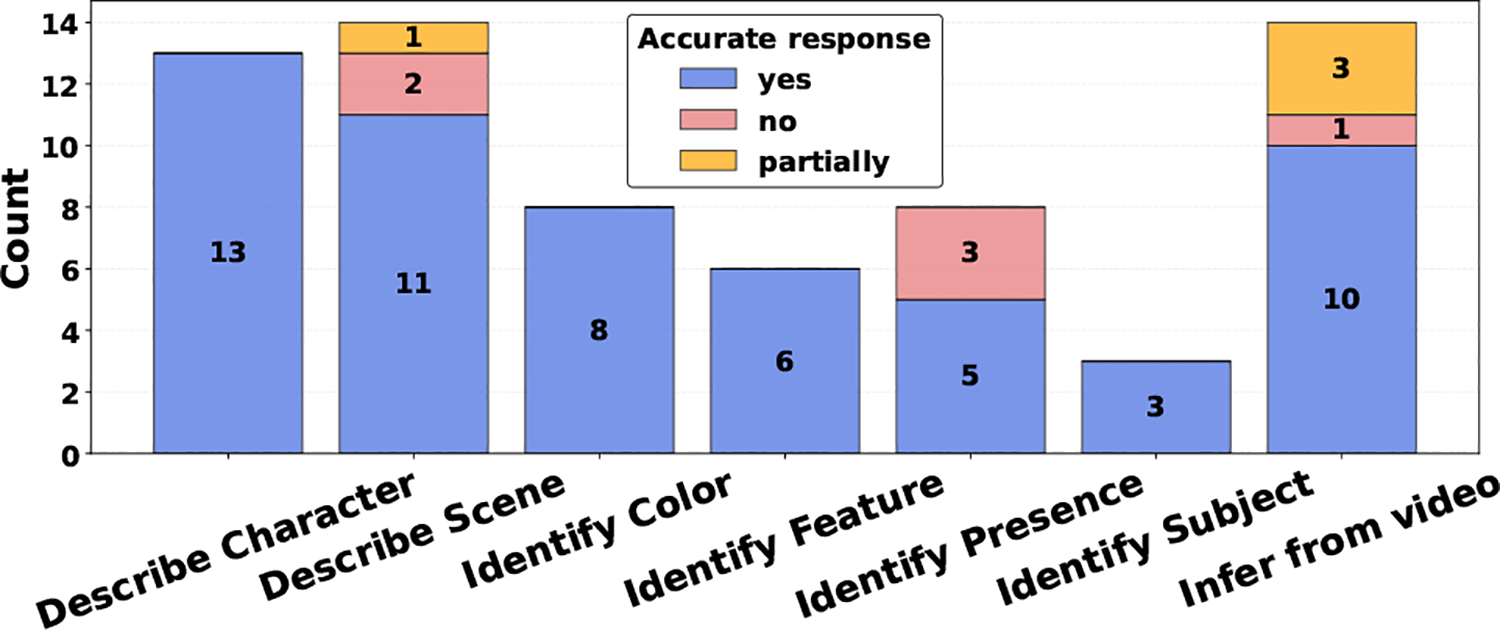
Distribution of VQA types and response accuracy.

Figure 3 shows the distribution of question types. Describing scene, character, and identifying color or presence were frequently asked questions. Several participants asked questions that required audio-visual inference (e.g., *Who said “I’m not on your team?”*), for which the VQA system sometimes failed to respond. In addition to these common patterns, participants reported meaningful use cases. For instance, P2 used VQA to obtain product-specific information (e.g., *What color is the cot?*), noting: *“I actually used this video and description [VQA] since I got one of these cots for Christmas.”*

### Longitudinal Changes.

Data showed notable patterns in how participants adjusted frequency and length parameters over time. Over subsequent days, participants increasingly opted for shorter description lengths, where the average reduced from 47.7 (*SD* = 21.0) to 33.3 (*SD* = 10.9). This trend aligns with qualitative feedback, where in the end of week survey, four participants explicitly reported a preference for reduced length: “*I liked the mid-length description, 25 words max. (P1)*” Participants indicated that higher word counts often introduced “AI fluff”. A similar pattern was observed for description frequency, with participants gradually favoring longer intervals (e.g., 15 seconds) as the week progressed to reduce interruptions and repetition. In contrast, no clear patterns emerged for emphasis or subjectiveness, suggesting these choices were primarily personal preference and content-driven.

Customization and VQA usage also remained consistent throughout the week. Specifically, perceived alignment of all customizations with the ADs remained high, with emphasis receiving the highest alignment rating (*M* = 4.22, *SD* = 0.56), followed by length (*M* = 4.10, *SD* = 0.70), frequency (*M* = 4.03, *SD* = 0.75), and subjectiveness (*M* = 3.97, *SD* = 0.55). Lower ratings for frequency and subjectiveness were due to perceived mismatches with user preferences, as P4 noted: *“Sometimes it felt like the AD wasn’t as frequent in the Mortal Kombat trailer. In the Kill Bill trailer, the subjective setting was too subjective.”* Together, these results suggest persistent perceptions across viewing sessions.

## 6 Discussion and Conclusion

We discuss implications of our findings for future work and outline the study’s limitations. Regarding the efficacy of MLLMs in AD customization (**RQ1**), our findings suggest participants have an overall positive perception of AI customizations in line with prior findings on customization by professional describers [24]. Our results also suggest high trust in the generated descriptions, but as the trust in AI increases, it raises concerns about correctness and hallucinations [20]. Prior research shows that users place high trust in longer descriptions regardless of their accuracy [14], a pattern that may similarly apply to customized descriptions. To mitigate such risks, AI accessibility systems can incorporate human-in-the-loop mechanisms and flag videos for review based on content or uncertainty in descriptions.

With respect to types of customization and questions (**RQ2**), *emphasis* emerged as the most preferred customization, with instructional emphasis favored for how-to, and character focus for entertainment content. Participants also often asked both scene-level and inferential questions. The combination of free-form guidelines and customization options in *ViDscribe* offers an opportunity to develop genre-specific AD guidelines that not only prompt MLLMs but also train novice describers. Also, the prevalence of inferential questions underscores the need for VQA systems that adopt a two-step approach: first, identifying relevant video segments, and then generating contextually grounded responses.

Regarding changes over time (**RQ3**), participants consistently used customizations and VQAs throughout the week. Our results suggest that shorter and less frequent descriptions were preferred over time. One possible explanation is that as participants became more familiar with the content style and system capabilities, they learned how much visual detail they could obtain relative to the number and frequency of ADs, leading them to favor more concise descriptions that reduced cognitive load and viewing interruption, similar to how professional describers shorten AI-generated or novice-written ADs [9]. Other customization choices varied by personal preference, as did the types of questions participants asked. These findings highlight a critical gap. While prior longitudinal data have examined BLV genre preferences [29], and interaction with image-QA [13], longitudinal engagement with AD customization and video-QA remains underexplored. Platforms like *ViDscribe* enable the collection of longitudinal data at scale, by capturing users’ customization choices and question-asking behavior over extended periods, generating rich behavioral data to inform adaptive systems that evolve ADs alongside users’ preferences.

### Limitations.

Although longitudinal, the one-week study duration may not be sufficient to capture longer-term adaptation of customizations. Additionally, to reduce participant burden, we opted to collect surveys (i.e., diary entries) at the end of each day rather than after each video. Gathering insights on individual video customizations could provide statistical evidence on the efficacy of AI customizations. Finally, the study involved eight BLV participants. Future work should look into how these customizations and VQA features scale across larger user populations.

In conclusion, *ViDscribe* enhances access and provides insights into user-driven customization and VQA usage, informing the design of future interactive AD systems.

## Supplementary Material

Supplemental

## Figures and Tables

**Table 1:** Six AD customization types and distribution of user-selected customizations.

Type	Option	n (%)	Type	Option	n (%)
**1. Emphasis**	general	27 (52.9)	**4.Color**	include	41 (80.4)
	character	15 (29.4)		exclude	10 (19.6)
	instructional	6 (11.8)	**5. Subjectivity**	objective	37 (72.5)
	environment	3 (5.9)		subjective	14 (27.5)
**2. Frequency**	8 seconds	28 (54.9)	**6. Length**	15–25 words	21 (41.2)
	15 seconds	22 (43.1)		26–50 words	25 (49.0)
	30 seconds	1 (2.0)		51–75 words	2 (3.9)
**3. Free-form Guidelines**		12 (23.5)		76–100 words	3 (5.9)

## CCS Concepts

• **Human-centered computing → Accessibility technologies**; **Accessibility systems and tools**; *User studies*; *Web-based interaction*.

## Footnotes

1
https://vidscribe.org/
